# ICH-LR2S2: a new risk score for predicting stroke-associated pneumonia from spontaneous intracerebral hemorrhage

**DOI:** 10.1186/s12967-022-03389-5

**Published:** 2022-05-04

**Authors:** Jing Yan, Weiqi Zhai, Zhaoxia Li, LingLing Ding, Jia You, Jiayi Zeng, Xin Yang, Chunjuan Wang, Xia Meng, Yong Jiang, Xiaodi Huang, Shouyan Wang, Yilong Wang, Zixiao Li, Shanfeng Zhu, Yongjun Wang, Xingquan Zhao, Jianfeng Feng

**Affiliations:** 1 Vascular Neurology, Department of Neurology, Beijing Tiantan Hospital, Capital Medical University, Beijing, 100070 China; 2 China National Clinical Research Center for Neurological Diseases, Beijing, China; 3 Institute of Science and Technology for Brain-Inspired Intelligence, Fudan University, Shanghai, 200433 China; 4 Chinese Institute for Brain Research, Beijing, China; 5 Research Unit of Artificial Intelligence in Cerebrovascular Disease, Chinese Academy of Medical Sciences, Beijing, China; 6 School of Computing, Mathematics and Engineering, Charles Sturt University, Albury, NSW 2640 Australia; 7 Ministry of Education, Key Laboratory of Computational Neuroscience and Brain-Inspired Intelligence (Fudan University), Shanghai, 200433 China; 8 MOE Frontiers Center for Brain Science and Shanghai Institute of Artificial Intelligence Algorithms, Fudan University, Shanghai, 200433 China; 9 Zhangjiang Fudan International Innovation Center, Shanghai, 200433 China

## Abstract

**Purpose:**

We develop a new risk score to predict patients with stroke-associated pneumonia (SAP) who have an acute intracranial hemorrhage (ICH).

**Method:**

We applied logistic regression to develop a new risk score called ICH-LR2S2. It was derived from examining a dataset of 70,540 ICH patients between 2015 and 2018 from the Chinese Stroke Center Alliance (CSCA). During the training of ICH-LR2S2, patients were randomly divided into two groups – 80% for the training set and 20% for model validation. A prospective test set was developed using 12,523 patients recruited in 2019. To further verify its effectiveness, we tested ICH-LR2S2 on an external dataset of 24,860 patients from the China National Stroke Registration Management System II (CNSR II). The performance of ICH-LR2S2 was measured by the area under the receiver operating characteristic curve (AUROC).

**Results:**

The incidence of SAP in the dataset was 25.52%. A 24-point ICH-LR2S2 was developed from independent predictors, including age, modified Rankin Scale, fasting blood glucose, National Institutes of Health Stroke Scale admission score, Glasgow Coma Scale score, C-reactive protein, dysphagia, Chronic Obstructive Pulmonary Disease, and current smoking. The results showed that ICH-LR2S2 achieved an AUC = 0.749 [95% CI 0.739–0.759], which outperforms the best baseline ICH-APS (AUC = 0.704) [95% CI 0.694–0.714]. Compared with the previous ICH risk scores, ICH-LR2S2 incorporates fasting blood glucose and C-reactive protein, improving its discriminative ability. Machine learning methods such as XGboost (AUC = 0.772) [95% CI 0.762–0.782] can further improve our prediction performance. It also performed well when further validated by the external independent cohort of patients (n = 24,860), ICH-LR2S2 AUC = 0.784 [95% CI 0.774–0.794].

**Conclusion:**

ICH-LR2S2 accurately distinguishes SAP patients based on easily available clinical features. It can help identify high-risk patients in the early stages of diseases.

**Supplementary Information:**

The online version contains supplementary material available at 10.1186/s12967-022-03389-5.

## Introduction

As the major complication of a stroke, stroke-associated infections (SAIs) have resulted in increased mortality [1]. It is reported that approximately 30% of post-stroke patients have infections [2]. Among those with infections, stroke-associated pneumonia (SAP), the most acute type of SAI, has the worst impact on functional outcomes [3, 4]. The incidence rate of SAP is approximately 10% among stroke patients but could be as high as 40% among high-risk populations [5]. Except for the relatively high incidence rate, SAP has serious consequences such as increased mortality, extended hospital stays, and deteriorated functional outcomes at discharge [6].

However, previous studies lacked effective prophylactic treatment for SAP in clinical practice [7, 8]. One of the reasons for such a failure of clinical trials of prophylactic antibiotics is the difficulty in selecting patients with the highest risk of SAP [9]. Consequently, clinical practice has no suitable routine measures to identify patients with the highest risk of developing SAP. Accurately selecting such patients could improve the results of future clinical trials. For prevention and treatment, it is, therefore, crucial to accurately identify those patients at risk during the acute phases of stroke.

SAP mainly includes acute ischemic stroke (AIS) and intracranial hemorrhage (ICH) stroke pneumonia. Rates of pneumonia are reportedly higher in patients with ICH than those with AIS [10]. However, most SAP research focuses on AIS, with relatively few studies examining ICH [11–13]. Therefore, it requires an objective and easily applicable model that predicts the probability of the development of pneumonia in ICH patients. Several recently developed clinical scores are available to predict SAP for stroke patients. Examples are the Pneumonia Score [14], Veteran’s Health Administration cohort score [15], ICH-APS (Intracerebral Haemorrhage-Associated Pneumonia Score) A and B [16], Pneumonia (PNA) prediction score [17], ISAN (Prestroke Independence, Sex, Age, NIHSS) score [18], ACDD4 (Age, Dysarthria, Dysphagia, CHF) [19], and PASS (Preventive Antibiotics in Stroke Study) [20]. Although most of these scores are SAP scoring models for AIS, some are also suited to predicting ICH patient scores [21]. According to previous studies [22, 23], SAP is associated with various risk factors, including older age, male gender, dysphagia, stroke-induced immunodepression syndrome, and chronic obstructive pulmonary disease (COPD). Different clinical scores consider different risk factors, with their comparisons detailed in Additional file 1: Table S1. Except for this, these studies on clinical scores have two main drawbacks. First, they rely on relatively small datasets ranging from 286 to 11,551 for predicting SAP (see Additional file 1: Table S2). Second, some variables used in these risk scores are not easily accessible directly. For example, a recent risk score for ICH, ICH-APS-B uses hematoma volume, infratentorial location, and extension into ventricles. As such, their conclusions may limit generalizability. Therefore, a new risk score should be developed for predicting ICH-associated SAP by using large-scale, multi-center data and clinical variables with readily available values.

In this study, we developed an ICH risk score called ICH-LR2S2 to predict pneumonia for risk assessment. It has been evaluated by the two large-scale multi-center cohorts. Except for the risk score, our machine learning model can be extended by adding additional variables to further improve the accuracy of its predictions. Note that this is a prospective study on predicting intracerebral hemorrhage stroke-associated pneumonia. More importantly, using the external independent validation data cohort has further demonstrated the benefits for clinical practice from our methods.

## Methods

### Participants

This study collected data on over one million patients from the Chinese Stroke Center Alliance (CSCA), a national, hospital-based, multi-center program initiated in August 2015. The CSCA requires participating hospitals to only enroll patients who meet the following criteria: (1) over 18 years old; (2) had the primary diagnosis of acute stroke/transient ischemic attacks (TIA) confirmed by brain CT or MRI, including acute AIS, TIA, intracerebral hemorrhage, or subarachnoid hemorrhage (SAH); (3) within seven days of symptom onset; and (4) admitted to hospital either directly or through emergency departments. Patients with cerebral venous sinus thrombosis or non-cerebrovascular diseases were excluded. For ensuring the accuracy of diagnosis and the quality of stroke care, performance metrics were used over the whole controlling process by strictly following the national standards and guideline recommendations prespecified or updated by the Steering Committee of CSCA. Detailed information about the CSCA design and methodology can be found in previous publications [24]. This study had been approved by the Central Institutional Review Board of Beijing Tiantan Hospital.

Patients with intracranial hemorrhagic stroke were selected, resulting in a total of 83,063 patients as our study cohort. Among the selected patients, 61,869 patients had no pneumonia (74.47%), while 21,194 patients had pneumonia (25.52%). There are more than 500 characteristic variables, including clinical variables on admission such as blood pressure, blood sugar, uric acid, pneumonia, National Institute of Health stroke scale (NIHSS), and modified Rankin Scale (mRS), as well as external variables such as hospital level, education level, and family income status.

### Definition and indicators of pneumonia

Pneumonia can be diagnosed by a typical chest X-ray, clinical symptoms, signs such as a cough, purulent sputum, fever, and laboratory tests such as white blood cell count. SAP after ICH can be diagnosed by a treating physician who uses clinical and laboratory indicators of respiratory infections such as fever, cough, and auscultation of respiratory cracks, new purulent sputum, or positive sputum culture, together with typical chest X-ray findings from PISCES (Pneumonia in Stroke ConsEnsuS) [25]. Hospital-acquired pneumonia was documented by excluding those cases that occurred before the stroke. Data on the development of SAP after ICH were prospectively collected.

### Study procedure

**Fig. 1:**
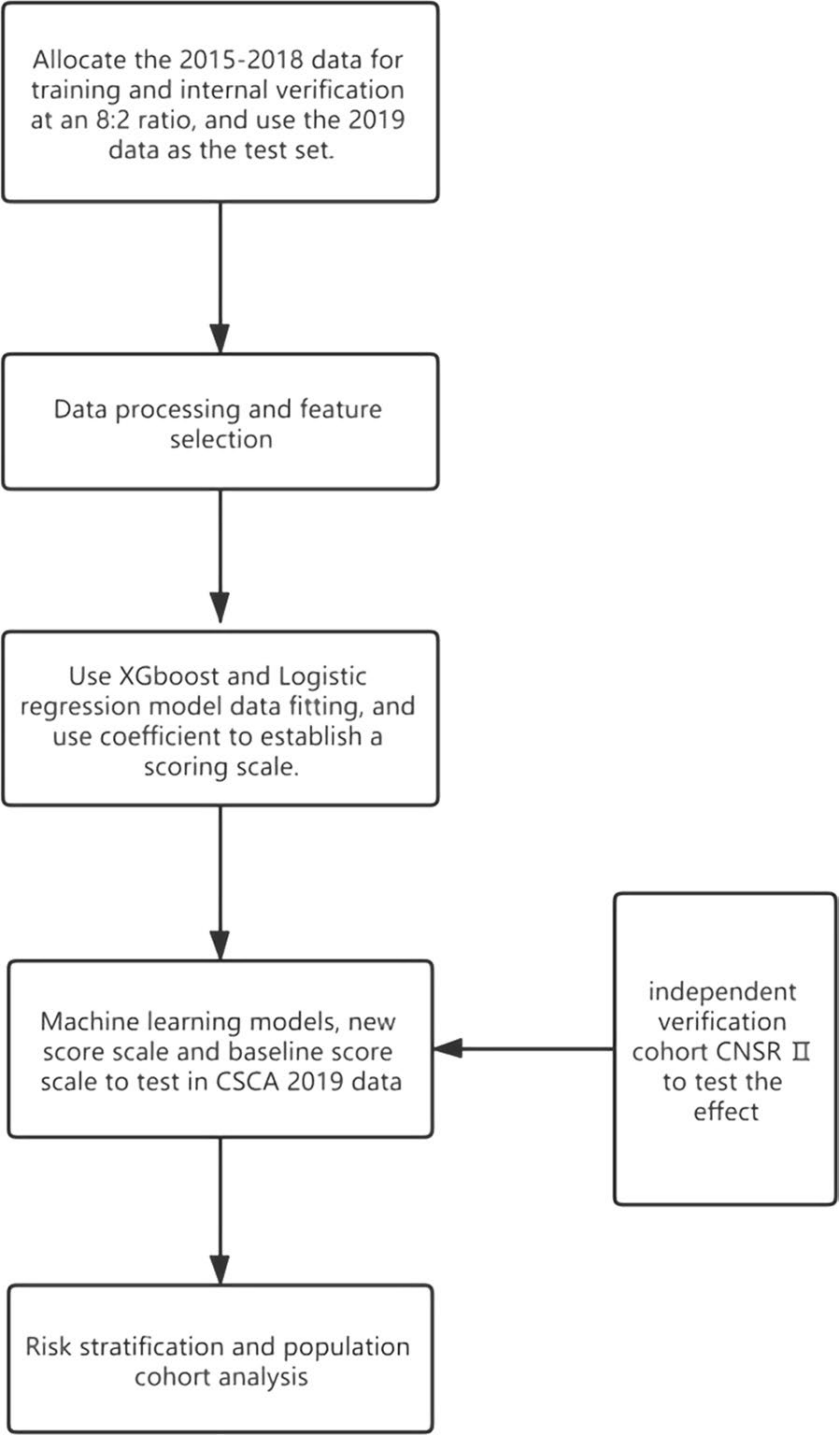
Flow diagram for the derivation cohort, validation cohort, and the schema of our model

To validate our model for predicting the likelihood of pneumonia, we used data collected from two multi-center cohorts in this study – the internal prospective research cohort of CSCA and the external independent verification cohort of CNSR II. In our experiments, we allocated the data from 2015 to 2018 for training with an internal verification ratio of 8:2, and the 2019 data for testing. After that, data records with missing values were filled in through data processing. The feature selection was then performed to select features that have important impacts on pneumonia. To this end, we first trained our model by using the two classic models—XGboost and logistic regression. ICH-LR2S2 was then calculated using the feature weight coefficients of logistic regression. After consulting doctors, the score interval was slightly modified according to the medical risk values to comply with the medical consensus [16, 26]. Additionally, we examined its performance on an external verification cohort. For the benefit of clinical practice, we stratified the patient population and analyzed the whole population cohort. The flow diagram is shown in Fig. 1.

### Data processing

Additional file 1: Table S3 shows the proportion of missing data for the selected variables. If a variable with a missing value was a continuous one, we filled it with the median value of that variable in the dataset. If it was binary, we filled it with 0, which means that there is no such disease history (our binary variables only include disease history and gender, and the gender variable is not missing). We finally obtained the data with the training set = 56,432, internal validation cohort = 14,108, and test set = 12,523 in our experiments.

### Feature selection

Considering medical variables from the perspective of clinical practice, we focused on screening medical variables related to human physiological characteristics and disease history conditions. We tried to select as few variables as possible without reducing the prediction accuracy for pneumonia. Feature selection was performed using the permutation method [27], which is suitable for tree models. The importance of a feature can be measured by how much the objective score decreases as a result of removing the feature. Specifically, the variable weights were calculated through the permutation mechanism provided by XGboost [28], which is a boosting tree model with the capacity to handle missing values. Ten-fold cross-validation tests on the training set were conducted to calculate the feature weights.

We filtered out the feature variables in turn, according to their weight order. A newly added feature must increase the overall score of the internal verification cohort by at least 0.005 in the cross-validation. Considering the features selected in the previous studies [16–21] as well as recommended by the doctors, we further added three new variables—gender, current smoking, and C-reactive protein. Finally, we ended up with 12 variables of dysphagia, Glasgow Coma Score (GCS), age, gender, fasting blood glucose, uric acid, COPD, National Institutes of Health Stroke Scale admission score (NIHSS score), mRS, current smoking, serum creatinine, and C-reactive protein. Detailed descriptions of these variables are provided in Additional file 1: Figure S3.

### Baseline scores

In Additional file 1: Tables S1 and S2, we list the scoring scales that can be used in ICH as the predictions of SAP in recent years. The aged variable is used by all scores, so is NIHSS except for the ACDD4 score [19]. In the following experiments, we mainly considered clinical variables that are easy to obtain. Therefore, we screened ICH-APS-A (from now on referred to as ICH-APS), PASS, ISAN, and PNA. It is worth mentioning that we made a compromise for ICH-APS; that is, we used drinking history instead of excessive drinking in ICH-APS. And considering the acquisition of variables, we set a score of 0 for the three medical variables (hematoma volume, infratentorial location, and extension into ventricles) that are not included in our data cohorts.

### ICH-LR2S2

We used classic machine learning models of logistic regression. Calculating the medical risk score by using the regression coefficient [29] and the prior medical consensus [16, 19, 21], we developed the ICH-LR2S2 risk score shown in Table 1. We excluded features with scores of less than one point. As such, ICH-LR2S2 used the nine patient features: age, mRS, fasting blood glucose, NIHSS score, GCS, C-reactive protein, dysphagia, COPD, and current smoking. Compared with previous risk scores, ICH-LR2S2 has two new variables—fasting blood glucose and C-reactive protein.

**Table 1:** ICH-LR2S2

Item	Range	Score
Age group	< 60	0
	60–69	1
	70–79	2
	80–89	3
	≥ 90	4
mRS	< 4	0
	4	2
	5	3
Fasting blood glucose	< 6	0
	6–8	1
	9–11	2
	≥ 12	3
NIHSS score	< 5	0
	5–13	1
	14–21	2
	22–29	3
	≥ 30	4
GCS	3–5	2
	6–8	1
	≥ 9	0
C-reactive protein	< 7	0
	7–16	1
	≥ 17	2
Dysphagia	Yes	4
	No	0
COPD	Yes	3
	No	0
Current smoking	Yes	2
	No	0

### External validation cohort

The performance of our model was tested on an independent cohort from the China National Stroke Registry II (CNSR II) [30]. As a nationwide initiative, the CNSR II, launched in 2012 by the Ministry of Health of China, established a reliable national stroke database for evaluating the delivery of stroke care in clinical practice. The CNSR II cohort included patients recruited from all 219 urban hospitals that voluntarily participated in the General Administration of Stroke Registration of China from June 2012 to January 2013. The study had been approved by the Central Institutional Review Board of Beijing Tiantan Hospital. Each participant provided written informed consent before participating.

### Statistical analysis

Continuous variables are described by means and standard deviations (SD), while categorical variables are described by counts and percentages. The prediction performances of the models are measured by the area under the receiver operating characteristic curve (AUC), with a 95% confidence interval (CI). The AUCs of these models were compared using the DeLong test [31]. The student’s *t*-test was used for continuous variables and the chi-square test for categorical variables. Two-sided p < 0.01 was considered to be statistically significant.

Based on logistic regression, the risk score used weight coefficients. By taking ten years as the interval, the ratio of a feature weight to the age weight coefficient was calculated to obtain the corresponding feature score and numerical interval. For a binary variable, the presence or absence of a feature was used as a scoring criterion (gender features give scores to men). For a continuous variable, in addition to considering the weight coefficient from the model, the actual meaning of the medical feature (medical risk range for this feature) must also be considered. In particular, the minimum unit of the score was 1 point, and features with less than 1 point were not scored. Based on the predictive score of the model, we stratified the risk of the population cohort and specified the risk threshold. We analyzed different risk groups by calculating the number of patients, the pneumonia rate, accuracy, sensitivity, specificity, PPV, and NPV (for more detailed information, refer to Additional file 1: Tables S7–S14).

## Results

### The composition and characteristics of the population

From July 2015 to June 2019, CSCA recruited 83,063 ICH patients, of which 21,194 (25.52%) were pneumonia patients. Specifically, 62.55% of all patients were male, with an average age of 62.48 years. For more detailed information, refer to Table 2. It can be found that there is a significant difference between the proportion of patients with dysphagia and pneumonia (36.63%) and those with dysphagia who did not have pneumonia (9.01%). This can also explain why the weight coefficient of this variable is large.

**Table 2:** Risk factors and basic knowledge in CSCA cohort (N: number of people)

Basic information	Total patients (n = 70,540)	With pneumonia (n = 18,190)	Without pneumonia (n = 52,350)	P values
Male (n%)	44,123 (62.55%)	11,864 (65.22%)	32,259 (61.62%)	< 0.01
Age (mean)	62.48	65.03	61.59	< 0.01
< 60 (N)	28,305	5908 (32.48%)	22,397 (42.783%)	< 0.01
60 ≤ age < 70 (N)	18,435	4636 (25.49%)	13,799 (26.36%)	0.01
70 ≤ age < 80 (N)	12,839	4,013 (22.06%)	8826 (16.86%)	< 0.01
80 ≤ age < 90 (N)	5644	2,170 (11.93%)	3474 (6.64%)	< 0.01
age ≥ 90 (N)	5317	1,463 (8.04%)	3854 (7.36%)	< 0.01
mRS at hospital (mean)	2.11	2.45	2.00	< 0.01
≤ 4 (N)	60,044	13,764 (75.67%)	46,280 (88.41%)	< 0.01
≥ 5 (5,6) (N)	10,496	4,426 (24.33%)	6,070 (11.60%)	0.02
NIHSS score (mean)	8.17	13.51	6.69	< 0.01
< 10 (N)	15,348	2061 (11.33%)	13,287 (25.38%)	< 0.01
10–16 (N)	2825	928 (5.10%)	1897 (3.62%)	0.03
> 16 (N)	2983	1523 (8.37%)	1460 (2.79%)	0.05
GCS (mean)	11.42	9.68	12.17	< 0.01
≥ 10 (N)	24,615	5527 (30.38%)	19,088 (36.46%)	< 0.01
< 10 (N)	10,847	5165 (28.40%)	5,682 (10.85%)	< 0.01
Smoking	14,165	3780 (20.78%)	10,385 (19.83%)	< 0.01
COPD	1,026	517 (2.84%)	509 (0.97%)	0.01
Dysphagia	11,379	6663 (36.63%)	4,716 (9.01%)	< 0.01
CRP (> 10 mg/l)	854	318 (1.75%)	536 (1.02%)	< 0.01
Creatinine (µmol/l)	83.16	86.37	82.06	< 0.01
Uric acid (µmol/l)	288.56	280.83	291.25	< 0.01
Fasting blood glucose (mmol/l)	6.54	7.00	6.38	< 0.01
< 7.8	56,617	13,421 (73.78%)	43,196 (82.51%)	< 0.01
7.8–11.1	8708	3059 (16.81%)	5649 (10.79%)	< 0.01
≥ 11.1	3805	1349 (7.42%)	2456 (4.69%)	0.02

The external validation cohort of CNSR II included 24,680 patients with similar demographic characteristics (male 63.75%, mean age 64.1 years, SD 12.0). Related surveys show that patients from the 2012 to 2013 dataset had insurance through new rural cooperative medical schemes [30]. These patients received better medical assistance and thus had a lower incidence of stroke and stroke-related complications. Additional file 1: Table S4 lists other characteristics of the derived cohort and external verification cohort. By comparing the data populations of the two cohorts, the proportion of pneumonia patients in CNSR II is only 8.44% and in CSCA it is 25.52%. We compared their representative characteristics. In medical judgment, a high NIHSS score, or a low GCS score indicates that a patient’s condition is serious. Patients with high scores for NIHSS variables (> 16) accounted for 4.93% in CNSR II, which was lower than in CSCA (13.18%). Patients with low scores for GCS variables (< 10) accounted for 1.45% in CNSR II, which was also lower than in CSCA (30.67%). For dysphagia, which has the greatest impact on the weight coefficient of pneumonia, its incidence rate in CNSR II was 8.29%, compared to 15.98% in CSCA. All these results demonstrate that the patients in the CNSR II data cohort had milder cases of pneumonia.

### Classification performance

The performance of the model was examined using the test data. The results of risk scores were as follows: ICH-LR2S2 (AUC = 0.749) [95% CI 0.739–0.759], the existing scoring method ICH-APS (AUC = 0.704) [95% CI 0.694–0.714], PASS (AUC = 0.684) [95% CI 0.674–0.694], ISAN (AUC = 0.676) [95% CI 0.666–0.686], and PNA (AUC = 0.636) [95% CI 0.626–0.646]. As a white-box model, ICH-LR2S2 is highly explanatory and intuitive, performing significantly better than the baseline of ICH-APS, PASS, and so on. The DeLong test results (see Additional file 1: Table S17) show that ICH-LR2S2 performed significantly better than other risk scores for ICH.

Compared with the previous scoring scales, we used newly added fasting blood glucose variables and C-reactive protein biomarkers in ICH-LR2S2. To validate this choice, we compared the effects of ICH-LR2S2 by deleting these two variables, respectively (see Additional file 1: Fig. S5 and S6). For the CSCA cohort data, AUC was reduced by 0.005 after removing fasting blood glucose, by 0.012 after removing C-reactive protein, and by 0.017 after removing both. For the external verification queue CNSR II, the AUC decreases by 0.009 after removing these two variables.

**Fig. 2:**
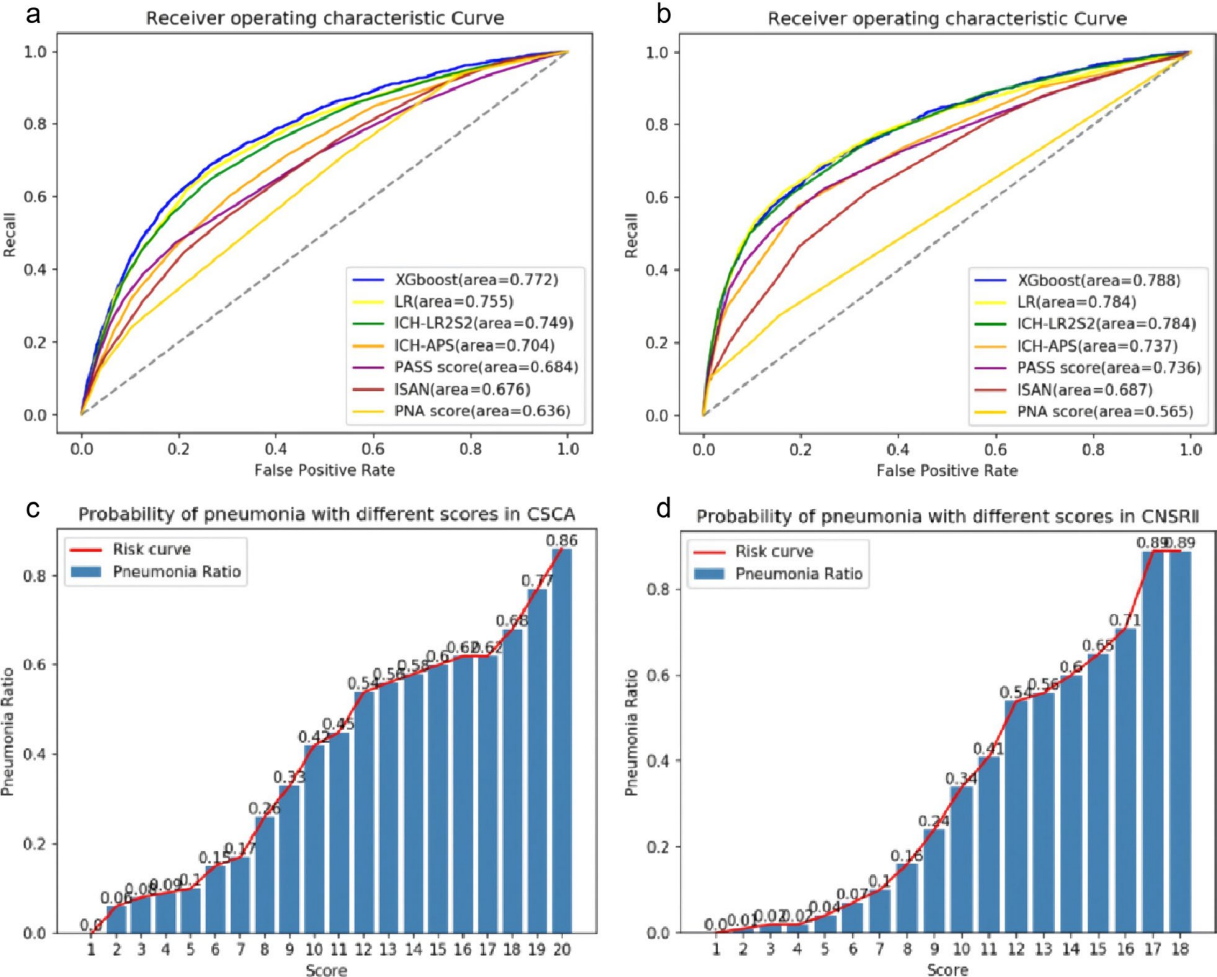
**a** Receiver operating characteristic curves (ROC) for discriminatory abilities of the different scores for clinical diagnosis of stroke-associated pneumonia (SAP) of CSCA cohort. **b** Receiver operating characteristic curves (ROC) for discriminatory abilities of the different scores for clinical diagnosis of stroke-associated pneumonia (SAP) of CNSR II cohort. **c** Probability of pneumonia with different scores in the CSCA cohort. **d** Probability of pneumonia with different scores in the CNSR II cohort

Furthermore, the machine learning model that uses more variables (gender, uric acid, and serum creatinine) shows better predictive performance in terms of comparing the risk score. As shown in Fig. 2(a), the results were as follows: XGboost (AUC = 0.772) [95% CI 0.762–0.782] and logistic regression (AUC = 0.755) [95% CI 0.745–0.765].

### External validation

We tested the risk score of ICH-LR2S2 on an independent external cohort of CNSR II. The CNSR II data statistics are reported in Additional file 1: Table S4. The results of external validation are consistent with those of test data. The overall performance of ICH-LR2S2 was AUC = 0.784 [95% CI 0.774–0.794] and the best performance of our baseline risk scoring models was ICH-APS (AUC = 0.737) [95% CI 0.727–0.747]. Also, our machine learning model can be further improved, as shown in Fig. 2b. The detailed results are reported in Additional file 1: Table S6, indicating that ICH-LR2S2 has high potential application values.

### Risk stratification

We divided the validation data set into different risk cohorts according to the given risk threshold. For the risk scoring scale, for example, we regarded people with a score higher than 13 as a high-risk group, people with a score lower than 6 as a low-risk group, and the rest as a medium-risk group. For the CSCA cohort, the number of patients in three risk groups were as follows: 556 (4.44%) in the high-risk group, with a pneumonia rate of 61.51%; 5659 (45.19%) in the middle-risk group, with a pneumonia rate of 33.89%; and 6308 (50.37%) in the low-risk group with a pneumonia rate of 11.79%. For XGboost, we also performed a risk stratification and cohort analysis based on predicted scores. The detailed results of the statistical analysis are given in Additional file 1: Tables S7–S14.

By using ICH-LR2S2, we listed the pneumonia probabilities in the table in terms of different scores (see Additional file 1: Tables S15 and S16). The bar graphs in Fig. 2c, d illustrate the corresponding relationships between ICH-LR2S2 scores and their probabilities of pneumonia. These figures provide doctors with an easy way of estimating a risk score in their clinical practice. For example, for the CSCA cohort, the probability of having pneumonia is 0.86 when the score reaches 20. As such, the information in this table can assist doctors in making decisions on patients with different conditions.

## Discussion

Previous studies have compared the different SAP scores in AIS by listing their pros and cons [26, 33]. Similarly, we compare the widely used SAP scores in ICH in Additional file 1: Table S1 from a different perspective. As demonstrated, the performance of our new risk score is superior to all existing scoring scales for predicting SAP of ICH. Further, the obvious advantage of ICH-LR2S2 lies in the use of widely available variables upon patients’ admissions. The corresponding score and pneumonia risk can then be easily calculated. More importantly, ICH-LR2S2 for physicians is a simple, intuitive, and easy-to-use pneumonia assessment model. Surprisingly, we discovered new variables that are influential on SAP in ICH, and the risk stratification based on our risk score corresponds to the probabilities of pneumonia from this study.

From this study, we concluded that fasting blood glucose levels and C-reactive protein play an important role in predicting SAP based on our large-scale data analysis. Both blood glucose level and diabetes history were regarded as important predictors in previous studies on SAP in AIS, but they were ignored in ICH [34]. Note that the history of diabetes does not represent abnormal blood glucose levels. Also, temporary hyperglycemia may indicate stress hyperglycemia rather than diabetes. As Hotter et al. [32] pointed out, diabetes history is not an independent risk factor for SAP; however, hyperglycemia reduces the bactericidal ability of white blood cells so that the possibility of lung infection increases, and patients with fasting hyperglycemia are likely to suffer from SAP [32, 33]. All these conclusions are consistent with our findings in this study. On the other hand, as an important biomarker, C-reactive protein (CRP) plays a significant role in predicting pneumonia [34]. Specifically, elevated CRP is an important sign of the poor prognosis of acute respiratory distress syndrome, reflecting the persistent state of inflammation [34, 35]. Adnet et al. [36] showed that high CRP levels help diagnose pneumonia patients with drug-induced coma and secondary inhalation. At the same time, they found that the sensitivity and specificity of other parameters (such as fever and white blood cell count) are poor indicators for diagnosing pneumonia. Based on our big data analysis, this study confirmed a strong correlation between CRP and SAP in ICH. Note that biomarkers and genetic data are needed to further investigate the mechanism of this correlation.

In terms of pneumonia in hemorrhagic stroke, it is clear from the data that patients with a history of hypertension are indeed more susceptible to pneumonia. Angiotensin can cause high blood pressure [37, 38], while high blood pressure is associated with a variety of diseases including stroke, diabetes, and so on [39]. As a drug, ARBs are one of the most commonly used first-line treatment drugs for hypertension [40]. By selectively blocking the angiotensin II receptor (AT1 type) and angiotensin II (Ang II), ARBs produce a pharmacological effect that is similar to that of angiotensin-converting enzyme inhibitors (ACEI) [41, 42]. They dilate blood vessels and lower blood pressure. Therefore, the use of ARB antihypertensive drugs can reduce the blood pressure of patients, which may reduce the probability of stroke and pneumonia in patients. In addition to ARB, it can be concluded from the data in this paper that the use of antihypertensive drugs, hypoglycemic drugs, and anticoagulants (see Additional file 1: Fig.S3) may be related to subsequent pneumonia infection in stroke patients. This warrants further research.

This study was conducted based on a large data registry collected from multi-centered hospitals in China. Compared to our CSCA study cohort, patients in the CNSR II cohort had fewer strokes and were less likely to have pneumonia. The statistical analysis of important characteristic indicators shows that the proportion of the population within the characteristic risk threshold is significantly smaller. This is due to the different admission times of patients and different periods of policies followed. Being validated by an external validation cohort, ICH-LR2S2 can still make accurate predictions on these datasets. This indicates that ICH-LR2S2 has good generalization capabilities. All validation results demonstrate that the variables we choose for ICH-LR2S2 can effectively distinguish those who do not have pneumonia from those who do.

For the stratification of patients at the greatest risk, we used machine learning and scoring methods to conduct risk stratification of the current patient population, and statistically analyzed various indicators of different risk population cohorts. For ICH-LR2S2, we plotted the histogram of the probabilities of pneumonia under different scores. We also tested the performance of risk stratification in an external verification cohort, and the results demonstrated its effectiveness in distinguishing different risk populations. With the same pneumonia risk rate (the error does not exceed 0.02) in the data, we compared our risk score ICH-LR2S2 with the two best-performing risk scores of ICH-APS and PASS in terms of population coverage in different risk cohorts. As shown in Additional file 1: Fig. S7 and S8, ICH-LR2S2 has a higher coverage in high-risk and low-risk populations, indicating that it can distinguish different risk population cohorts better than ICH-APS and PASS. Specifically, in the CSCA cohort, the population coverage rate for the high-risk cohort was 4.44% by ICH-LR2S2, which was higher than 3.08% by ICH-APS and 2.89% by PASS. ICH-LR2S2 has also demonstrated consistent performance on different datasets (see Additional file 1: Fig. S9 and S10). With such good performance, ICH-LR2S2 can be used to score the risk of a new patient in clinical practice. A patient can be classified into the corresponding risk population cohort by using ICH-LR2S2. As such, medical expenses can be saved. Further, different post-response measures can be adopted for targeted treatment and care. On the other hand, several factors should be considered before using our ICH-LR2S2. For example, the threshold in ICH-LR2S2 should be set based on the different incidences of SAP or a hospital’s clinical priorities. Factors such as the acceptability of risks or the adoption of reasonable precautions may also affect the choice of a particular threshold.

By using the limited number of scoring variables, clinical risk scoring can be simplified at the expense of compromising the accuracy of prediction. For example, ICH-LR2S2 does not use the variables and factors of medical comorbidities, additional imaging data or additional medication history information such as anticoagulants or antibiotics. However, the prediction from our scores should be combined with the use of any other clinically relevant information. In other words, ICH-LR2S2 is intended for use as a tool to aid a clinical decision-making process. Moreover, future efforts will be geared toward investigating its best use. The experiment results also have indicated that ICH-LR2S2 is a valuable tool to help identify clinicians’ triage patients with ICH by predicting their risks of pneumonia reliably and accurately. In other words, our score can identify high-risk ICH patients who require additional interventional treatment.

## Limitations

First, stroke cohorts other than CSCA and CNSR can be selected for further validation. Second, the data lacks variables such as hematoma volume, submeningeal location, and intraventricular hemorrhage, making ICH-APS slightly less effective. Finally, ICH-LR2S2 is a model for predicting pneumonia risk. This model can be transferred to other types of stroke complication prediction. Its application and prognostic model in daily clinical practice remain to be studied.

## Conclusion

In this paper, we have presented ICH-LR2S2, which can accurately predict pneumonia associated with spontaneous intracranial hemorrhage by using data-driven machine learning methods. ICH-LR2S2 can be used easily by physicians of varying specialties.

## Supplementary Information

**Additional file 1.**

## Data Availability

The datasets used and/or analyzed in the current study are available from the corresponding author upon reasonable request.
