# Supplementary material for: ICH-LR2S2: a new risk score for predicting stroke-associated pneumonia from spontaneous intracerebral hemorrhage
Source: J Transl Med. 2022 May 4;20:193. doi: 10.1186/s12967-022-03389-5 (PMC9066782; doi:10.1186/s12967-022-03389-5)
Supplement: Supplementary file 1 — Additional file 1. [file 12967_2022_3389_MOESM1_ESM.docx]

**Appendix**


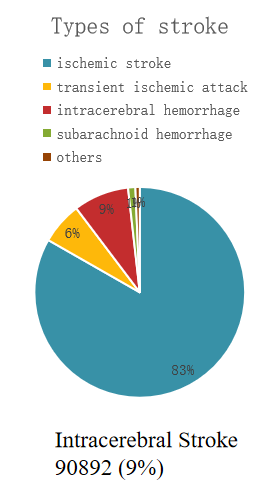


Figure S1: Proportions of diﬀerent types of stroke.


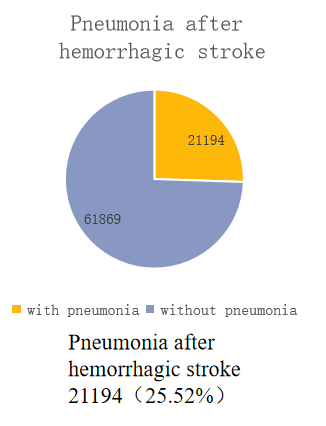


Figure S2: Proportions of Intracranial hemorrhagic stroke with pneumonia.

Table S1: Comparisons of pneumonia prediction scores. Different predictors are used to calculating the scores, where (✓) indicates a predictor used by the corresponding scores, such as age, which is used by all the scores. Abbreviations: NIHSS --National Institute of Health Stroke Scale, COPD --chronic obstructive pulmonary disease, mRS-- modified Rankin Scale, BP-- blood pressure, and CHF-- congestive heart failure.

| **Scale Score** | **ICH-LR2S2** | **ICH-APS-A** | **ICH-APS-B** | **pneumonia score** | **VHA** | **PNA** | **ACDD4** | **PASS** | **ISAN** |
| --- | --- | --- | --- | --- | --- | --- | --- | --- | --- |
| Age | √ | √ | √ | √ | √ | √ | √ | √ | √ |
| NIHSS | √ | √ | √ | √ | √ | √ |  | √ | √ |
| Dysarthria |  |  |  |  |  |  | √ |  |  |
| Dysphagia | √ | √ | √ | √ |  |  | √ | √ |  |
| Diabetes |  |  |  |  |  | √ |  |  |  |
| Atrial Fibrillation |  |  |  |  |  | √ |  |  |  |
| Sex |  |  |  | √ |  |  |  | √ | √ |
| Smoking | √ | √ | √ |  |  |  |  |  |  |
| COPD | √ | √ | √ |  |  |  |  | √ |  |
| Pre-stroke mRS | √ | √ | √ |  |  |  |  | √ | √ |
| Mechanical Ventilation |  |  |  | √ |  |  |  |  |  |
| GCS | √ | √ |  |  |  |  |  |  |  |
| CHF (congestive heart failure) |  |  |  |  |  |  | √ |  |  |
| Fasting blood glucose | √ |  |  |  |  |  |  |  |  |
| C-reactive protein | √ |  |  |  |  |  |  |  |  |
| Drinking |  | √ | √ |  |  |  |  |  |  |
| Hematoma volume(ml) |  |  | √ |  |  |  |  |  |  |
| infratentorial location |  | √ | √ |  |  |  |  |  |  |
| Extension into ventricles |  | √ |  |  |  |  |  |  |  |
| intracerebral hemorrhage |  |  |  |  | √ |  |  | √ |  |
| Abnormal swallowing test |  |  |  |  |  |  |  |  |  |
| Found down at symptom onset |  |  |  |  | √ |  |  |  |  |
| Past medical history of pneumonia |  |  |  |  | √ |  |  |  |  |

Table S2. Comparisons of models on predicting pneumonia or infection in intracerebral haemorrhage patients in terms of their data sizes for validation. RCT: Randomized Controlled Trial; RC: Retrospective cohort; PCS: Prospective cohort study; R: Registry. -: unavailable

| **Scale score** | **ICH-LR2S2** | **ICH-APS-A** | **ICH-APS-B** | **pneumonia score** | **VHA** | **PNA** | **ACDD4** | **PASS** | **ISAN** |
| --- | --- | --- | --- | --- | --- | --- | --- | --- | --- |
| Derivation  cohort size | 56,432 | 2,998 | 2,998 | 286 | 925 | 568 | 1,644 | 1,268 | 11,551 |
| Internal Validation cohort size | 14,108 | 2000 | 2000 | - | 438 | - | - | 1,270 | - |
| External validation cohort size | 24,860 | - | - | - | - | - | - | - | 11,648 |
| Study design | PCS | R | R | Cohort study | RC | RC | RC | RCT | R |

Table S3: Missing Data

| Variable | Missing data |
| --- | --- |
| C-reactive protein | 66,841 |
| NIHSS Score | 47,954 |
| GCS | 35,078 |
| Dysphagia | 20,644 |
| COPD | 7,316 |
| Fasting blood glucose | 1,108 |
| Uric acid | 1,117 |
| Serum creatinine | 677 |
| Age | 0 |
| Gender | 0 |
| mRS | 0 |
| Current smoking | 0 |

Table S4. Risk factors and basic knowledge of risk prediction of intracranial hemorrhage-associated pneumonia and the influence of intracranial hemorrhage-associated-pneumonia on clinical outcomes in the CSCA and CNSR Ⅱ cohorts.

| Basic  information | Train set in CSCA (n=56,432) | Valid set in CSCA (n=14,108) | Test set in CSCA (n=12,523) | CNSR Ⅱ (n=24,860) |
| --- | --- | --- | --- | --- |
| Pneumonia (n%) | 14,533(25.75%) | 3,657(25.92%) | 3,004(23.99%) | 2,097(8.44%) |
| Male, (n%) | 35,324(62.60%) | 8,799(62.37%) | 7,931(63.33%) | 15,600(62.75%) |
|  |  |  |  |  |
| Age (mean (SD)) | 62.47(12.60) | 62.51(12.63) | 63.07(12.62) | 64.10(11.99) |
| <60 (number of people) | 22,624(40.09%) | 5,681(40.27%) | 4,855(38.77%) | 8,766(35.27%) |
| 60≤age<70 (number of people) | 14,819(26.26%) | 3616(25.63%) | 3,305(26.39%) | 6,250(25.14%) |
| 70≤age<80 (number of people) | 10,259(18.18%) | 2,580(18.29%) | 2,389(19.08%) | 5,774(23.23%) |
| 80≤age<90 (number of people) | 4,507(7.99%) | 1,137(8.06%) | 1,115(8.90%) | 1,943(7.82%) |
| age≥90 (number of people) | 4,223(7.48%) | 1,094(7.75%) | 859(6.86%) | 2,127(8.68%) |
|  |  |  |  |  |
| mRS at hospital (mean (SD)) | 2.11(1.58) | 2.10(1.57) | 2.30(1.67) | 0.70(1.19) |
| ≤4 (number of people) | 48,011(85.08%) | 12,033(85.29%) | 10,321(82.42%) | 24,465(98.41%) |
| ≥5 (5,6) (number of people) | 8,421(14.92%) | 2,075(14.71%) | 2,202(17.58%) | 395(1.59%) |
|  |  |  |  |  |
| NIHSS score (mean (SD)) | 8.15(8.37) | 8.27(8.50) | 9.13(9.54) | 5.08(5.91) |
| <10 (number of people) | 12,296(68.21%) | 3,052(66.93%) | 4,241(64.19%) | 73,731(84.15%) |
| 10-16 (number of people) | 2,211(12.27%) | 614(13.47%) | 797(12.06%) | 632(7.20%) |
| >16 (number of people) | 2,376(13.18%) | 607(13.31%) | 1,105(16.72%) | 432(4.93%) |
|  |  |  |  |  |
| GCS (mean) | 11.42(4.11) | 11.43(4.10) | 11.42(4.11) | 13.89(1.02) |
| ≥10 (number of people) | 18,304(64.38%) | 4,561(64.89%) | 4,995(64.60%) | 24,414(98.21%) |
| <10 (number of people) | 8,721(30.67%) | 2,126(30.25%) | 2,360(30.52%) | 361(1.45%) |
|  |  |  |  |  |
| Smoking | 11,297(20.02%) | 2,868(20.33%) | 2,314(18.48%) | 0 |
| COPD | 811(1.60%) | 215(1.70%) | 186(1.63%) | 275(1.11%) |
| Dysphagia | 9,021(15.98%) | 2,358(16.71%) | 1,909(15.24%) | 2,062(8.29%) |
| CRP (>10mg/l) | 662(1.12%) | 192(1.36%) | 2,294(21.40%) | 1,161(4.67%) |
| Creatinine (µmol/l) | 82.98(82.56) | 83.89(87.43) | 82.43(82.51) | 75.60(44.27) |
| Uric acid (µmol/l) | 288.31(137.53) | 289.53(132.83) | 285.04(131.33) | 300.90(104.40) |
|  |  |  |  |  |
| Fasting blood glucose (mmol/l) | 6.54(2.83) | 6.54(2.84) | 6.59(2.77) | 6.32(2.54) |
| <7.8 | 45,249(81.50%) | 11,368(81.73%) | 9,989(80.95%) | 19,831(83.35%) |
| 7.8-11.1 | 6,981(12.57%) | 1,721(12.42%) | 1,575(12.76%) | 2,505(10.53%) |
| ≥11.1 | 3,051(5.50%) | 754(5.42%) | 7,28(5.90%) | 1,376(5.78%) |


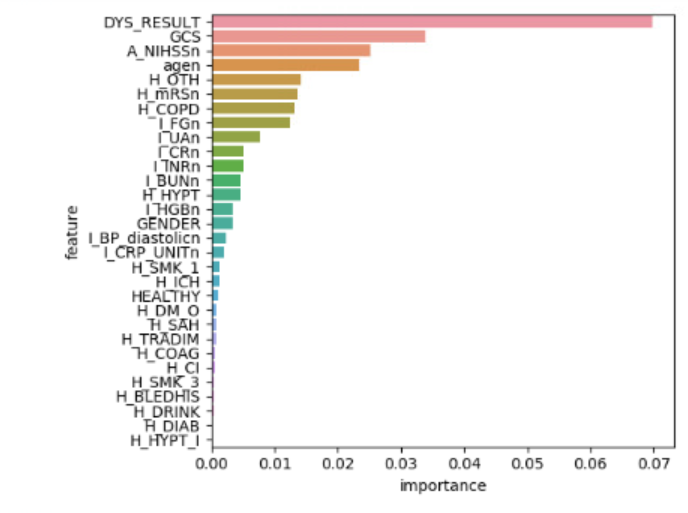


Figure S3: The meanings of variables, **DYS_RESULT**: dysphagia; **GCS:** Glasgow Score; **A_NIHSSn**: Visiting NIHSSn Score; **agen**: age; **H_OTH**: the history of other diseases; **H_mRSn**: modified Rankin Scale(mRS); **H_COPD**: Chronic obstructive pulmonary disease; **I_FGn**: Fasting blood glucose; **I_UAn**: Uric acid; **I_CRn**: Serum creatinine; **I_INRn**: INR value; **I_BUNn**: Serum urea nitrogen; **H_HYPT**: Antihypertensive drugs; **I_HGBn**: Glycated hemoglobin; **I_BP_diastolicn**: Diastolic blood pressure; **I_CRP_UNITn**: C-reactive protein; **H_SMK_1**: Never smoked; **H_ICH**=Cerebral hemorrhage; **HEALTHY**: Past ﬁtness; **H_DM_O**: Hypoglycemic drugs; **H_SAH**: Subarachnoid hemorrhage; **H_TRADIM**: Chinese medicine; **H_COAG**: Anticoagulant drugs; **H_CI**: Cerebral infarction; **H_SMK_3**: current smoking; **H_BLEDHIS**: Bleeding history or tendency; **H_DRINK**: Drinking history; **H_DIAB**: diabetes; **H_HYPT_I**: Hypertension.


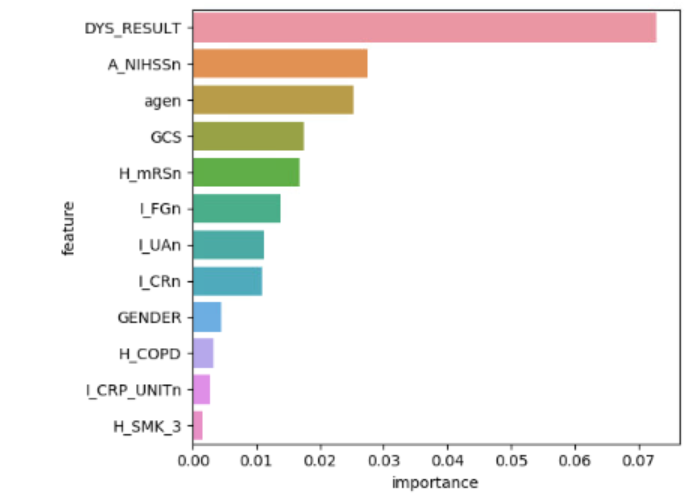


Figure S4: Permutation importance in our select variables.

Table S5. AUC scores of the machine learning models and score scales in intracranial hemorrhagic stroke pneumonia after data processing.

| **Model** | **AUC [95% confidence interval]** |
| --- | --- |
| PNA | 0.636 [0.626-0.646] |
| ISAN | 0.676 [0.666-0.686] |
| PASS | 0.684 [0.674-0.694] |
| ICH-APS | 0.704 [0.694-0.714] |
| ICH-LR2S2 | 0.749 [0.739-0.759] |
| Logistic Regression | 0.755 [0.745-0.765] |
| XGboost | 0.772 [0.762-0.782] |

Table S6: Model results for CNSR II cohort.

| **Model** | **AUC [95% confidence interval]** |
| --- | --- |
| PNA | 0.565 [0.555-0.575] |
| ISAN | 0.687 [0.677-0.697] |
| PASS | 0.736 [0.726-0.746] |
| ICH-APS | 0.737 [0.727-0.747] |
| Logistic Regression | 0.784 [0.774-0.794] |
| ICH-LR2S2 | 0.784 [0.774-0.794] |
| XGboost | 0.788 [0.778-0.798] |


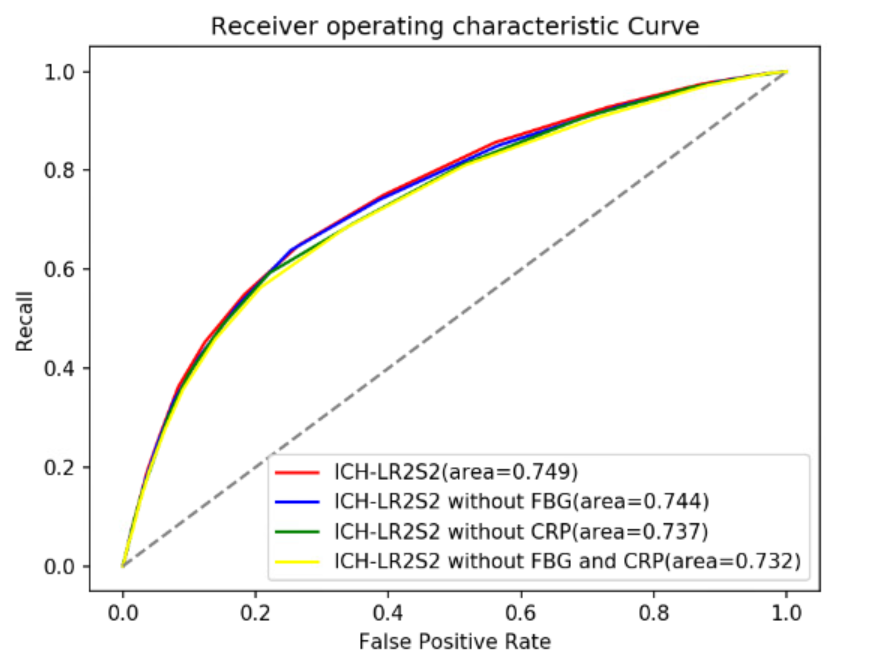


Figure S5: Comparing the ROC curve of ICH-LR2S2 and ICH-LR2S2 without using fasting blood glucose and C-reactive protein in CSCA. FBG, fasting blood glucose; CRP, C-reactive protein.


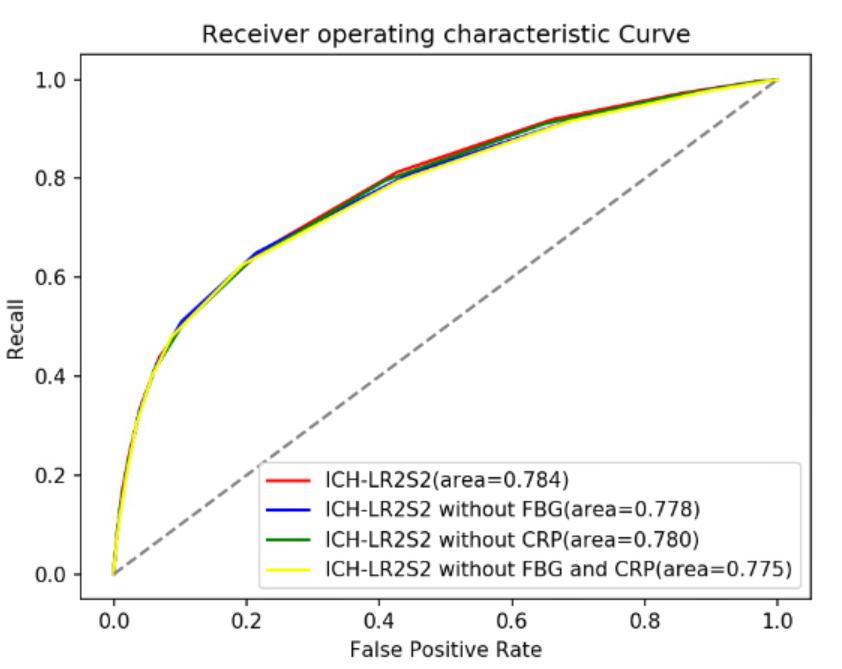


Figure S6: Comparing the ROC curve of ICH-LR2S2 and ICH-LR2S2 without using fasting blood glucose and C-reactive protein in CNSR II. FBG, fasting blood glucose; CRP,C-reactive protein.

Table 7: Risk stratification of CSCA cohort by XGboost. The threshold for identifying high-risk populations in XGboost was set to 0.70, accounting for 2.84% of the population, and 70.38% of people suffering from pneumonia. The threshold in XGboost for identifying low-risk populations was 0.15, accounting for 49.87% of the population, and those suffering from pneumonia occupying 10.06%. The areas with medium risk were divided into 0.15-0.70, accounting for 47.19% of the population, and those suffering from pneumonia account for 35.83%.

| Groups | Threshold | Number of patients | Pneumonia rate |
| --- | --- | --- | --- |
| High Risk | >0.70 | 368(2.84%) | 70.38% |
| Intermediate Risk | 0.15-0.70 | 5,909(47.19%) | 35.83% |
| Low Risk | <0.15 | 6,246(49.87%) | 10.06% |

Table 8: Risk stratification of CSCA cohort by XGboost. The XGboost threshold identifying high-risk was 0.70, with an Accuracy of 0.772 (0.762- 0.782), Sensitivity of 0.086 (0.076-0.096), Speciﬁcity of  0.988 (0.982-0.994), PPV of 0.703 (0.693-0.713), NPV of 0.774 (0.764-0.784); Low-risk threshold was 0.15, with an Accuracy of 0.638 (0.628-0.648), Sensitivity of 0.790 (0.780-0.800), Speciﬁcity of 0.590 (0.580-0.600), PPV of 0.378 (0.368-0.388), NPV of 0.899 (0.889-0.909).

| Groups | Threshold | Accuracy (95% CI) | Sensitivity (95% CI) | Speciﬁty (95% CI) | PPV (95% CI) | NPV (95% CI) |
| --- | --- | --- | --- | --- | --- | --- |
| High Risk | 0.70 | 0.772 (0.762-0.782) | 0.086 (0.076-0.096) | 0.988 (0.982-0.994) | 0.703 (0.693- 0.713) | 0.774 (0.764- 0.784) |
| Low Risk | 0.15 | 0.638 (0.628-0.648) | 0.790 (0.780-0.800) | 0.590 (0.580-0.600) | 0.378 (0.368- 0.388) | 0.899 (0.889- 0.909) |

Table S9: Risk stratification of CNSR II cohort by XGboost. The threshold used by XGboost for identifying high-risk populations is 0.70, accounting for 4.18% of the population, and 68.27% of people suffering from pneumonia; XGboost’s threshold for identifying low-risk populations is 0.15, accounting for 71.60% of the population, and those suffering from pneumonia occupying 3.76%; areas with the medium risk were divided into 0.15-0.70, accounting for 27.98% of the population, and those suffering from pneumonia account for 19.50%.

| Groups | Threshold | Number of patients | Pneumonia rate |
| --- | --- | --- | --- |
| High Risk | >0.70 | 104(4.18%) | 68.27% |
| Intermediate Risk | 0.15-0.70 | 6,956(27.98%) | 19.50% |
| Low Risk | <0.15 | 17,800(71.60%) | 3.76% |

Table S10: Risk stratification of CNSR II cohort by XGboost. The XGboost threshold for identifying high-risk was 0.70, with an Accuracy of 0.917 (0.907- 0.927), Sensitivity of 0.034 (0.024-0.044), Specificity of  0.998 (0.997-0.999), PPV of 0.683 (0.673-0.693), NPV of 0.918 (0.908-0.928); Low-risk threshold was 0.15, with an Accuracy of 0.747 (0.737-0.757), Sensitivity of 0.681 (0.671-0.691), Specificity of 0.753 (0.743-0.763), PPV of 0.202 (0.192-0.212), NPV of 0.962 (0.952-0.972).

| Groups | Threshold | Accuracy (95% CI) | Sensitivity (95% CI) | Speciﬁty (95% CI) | PPV (95% CI) | NPV (95% CI) |
| --- | --- | --- | --- | --- | --- | --- |
| High Risk | 0.70 | 0.917 (0.907-0.927) | 0.034 (0.024-0.044) | 0.998 (0.997-0.999) | 0.683 (0.673-0.693) | 0.918 (0.908-0.928) |
| Low Risk | 0.15 | 0.747 (0.737-0.757) | 0.681 (0.671-0.691) | 0.753 (0.743-0.763) | 0.202 (0.192-0.212) | 0.962 (0.952-0.972) |

Table S11: Risk stratification of CSCA cohort by ICH-LR2S2. The threshold for ICH-LR2S2 to identify high-risk populations was 13, accounting for 4.44% of the population, and 61.51% of people suffering from pneumonia; The threshold for identifying low-risk populations was 6, accounting for 50.37% of the population, and those suffering from pneumonia occupying 11.79%; areas with medium risk were divided into 6-13, accounting for 45.19% of the population, and those suffering from pneumonia account for 33.89%.

| Groups | Threshold | Number of patients | Pneumonia rate |
| --- | --- | --- | --- |
| High Risk | >13 | 556 (4.44%) | 61.51% |
| Intermediate Risk | 6-13 | 5,659 (45.19%) | 33.89% |
| Low Risk | <6 | 6,308 (50.37%) | 11.79% |

Table S12: Risk stratification of CSCA cohort by ICH-LR2S2. The ICH-LR2S2 threshold identifying high-risk 13, with an Accuracy of 0.770 (0.760-0.780), Sensitivity of 0.114 (0.106-0.122), Specificity of 0.977 (0.967-0.987), PPV of 0.615 (0.605-0.625), NPV of 0.778 (0.768-0.788); Low-risk threshold was 6, with an Accuracy of 0.624 (0.614-0.634), Sensitivity of 0.752 (0.742-0.762), Speciﬁcity of 0.585 (0.575-0.595), PPV of 0.364 (0.354-0.374), NPV of 0.882 (0.872-0.892).

| Groups | Threshold | Accuracy (95% CI) | Sensitivity (95% CI) | Speciﬁty (95% CI) | PPV (95% CI) | NPV (95% CI) |
| --- | --- | --- | --- | --- | --- | --- |
| High Risk | 13 | 0.770 (0.760-0.780) | 0.114 (0.106-0.122) | 0.977 (0.967-0.987) | 0.615 (0.605- 0.625) | 0.778 (0.768- 0.788) |
| Low Risk | 6 | 0.624 (0.614-0.634) | 0.752 (0.742-0.762) | 0.585 (0.575-0.595) | 0.364 (0.354- 0.374) | 0.882 (0.872- 0.892) |

Table S13: Risk stratification of CNSR II cohort by ICH-LR2S2. The threshold used by ICH-LR2S2 for identifying high-risk populations was 13, accounting for 0.45% of the population, and 65.18% of people suffering from pneumonia; The threshold for identifying low-risk populations was 6, accounting for 83.04% of the population, and those suffering from pneumonia occupying 4.59%; The areas with the medium risk were divided into 6-13, accounting for 16.71% of the population, and those suffering from pneumonia account for 25.95%.

| Groups | Threshold | Number of patients | Pneumonia rate |
| --- | --- | --- | --- |
| High Risk | >13 | 112 (0.45%) | 65.18% |
| Intermediate Risk | 6-13 | 4,124 (16.71%) | 25.95% |
| Low Risk | <6 | 20,494 (83.04%) | 4.59% |

Table S14: Risk stratification of CNSR II cohort by ICH-LR2S2. The ICH-LR2S2 threshold for identifying high-risk 13, with an Accuracy of 0.917 (0.907-0.927), Sensitivity of 0.034 (0.024-0.044), Speciﬁcity of 99.8 (0.997-0.998), PPV of  0.651 (0.641-0.661), NPV of  0.928 (0.918-0.938); Low-risk threshold was 6, with an Accuracy of 0.836 (0.826-0.846), Sensitivity of 0.549 (0.539-0.559), Speciﬁcity of 0.863 (0.853-0.873), PPV of 0.270 (0.260-0.280), NPV of 0.954 (0.944-0.964).

| Groups | Threshold | Accuracy (95% CI) | Sensitivity (95% CI) | Speciﬁty (95% CI) | PPV (95% CI) | NPV (95% CI) |
| --- | --- | --- | --- | --- | --- | --- |
| High Risk | 13 | 0.917 (0.907-0.927) | 0.034 (0.024-0.044) | 0.998 (0.997-0.999) | 0.651 (0.641- 0.661) | 0.928 (0.918- 0.938) |
| Low Risk | 6 | 0.836 (0.826-0.846) | 0.549 (0.539-0.559) | 0.863 (0.853-0.873) | 0.270 (0.260- 0.280) | 0.954 (0.944- 0.964) |

Table S15: ICH-LR2S2 predicts the probabilities of pneumonia.

| Score | 1 | 2 | 3 | 4 | 5 | 6 | 7 | 8 | 9 | 10 |
| --- | --- | --- | --- | --- | --- | --- | --- | --- | --- | --- |
| Ratio | 0 | 0.064 | 0.079 | 0.094 | 0.100 | 0.147 | 0.172 | 0.259 | 0.333 | 0.418 |
| Score | 11 | 12 | 13 | 14 | 15 | 16 | 17 | 18 | 19 | 20 |
| Ratio | 0.447 | 0.542 | 0.557 | 0.579 | 0.598 | 0.617 | 0.618 | 0.684 | 0.770 | 0.857 |

Table S16: ICH-LR2S2 predicts the probabilities of pneumonia for CNSR II cohort.

| Score | 1 | 2 | 3 | 4 | 5 | 6 | 7 | 8 | 9 | 10 |
| --- | --- | --- | --- | --- | --- | --- | --- | --- | --- | --- |
| Ratio | 0 | 0.009 | 0.023 | 0.024 | 0.035 | 0.068 | 0.095 | 0.161 | 0.240 | 0.343 |
| Score | 11 | 12 | 13 | 14 | 15 | 16 | 17 | 18 | 19 | 20 |
| Ratio | 0.412 | 0.535 | 0.556 | 0.595 | 0.650 | 0.706 | 0.889 | 0.889 | - | - |

Table S17: For the CSCA test set, Delong was used to test the model significance of the AUCs of different models.

|  | XGboost | Logistic Regression | ICH-LR2S2 | ICH-APS | PASS | ISAN | PNA |
| --- | --- | --- | --- | --- | --- | --- | --- |
| XGboost | 1 | <0.01 | <0.01 | <0.01 | <0.01 | <0.01 | <0.01 |
| Logistic Regression | <0.01 | 1 | 0.04 | <0.01 | <0.01 | <0.01 | <0.01 |
| ICH-LR2S2 | <0.01 | 0.04 | 1 | <0.01 | <0.01 | <0.01 | <0.01 |
| ICH-APS | <0.01 | <0.01 | <0.01 | 1 | 0.49 | <0.01 | <0.01 |
| PASS | <0.01 | <0.01 | <0.01 | 0.49 | 1 | 0.11 | <0.01 |
| ISAN | <0.01 | <0.01 | <0.01 | <0.01 | 0.11 | 1 | <0.01 |
| PNA | <0.01 | <0.01 | <0.01 | <0.01 | <0.01 | <0.01 | 1 |

Table S18: For the CNSR Ⅱ cohort, Delong was used to test the model significance of the AUCs of different models.

|  | XGboost | Logistic Regression | ICH-LR2S2 | ICH-APS | PASS | ISAN | PNA |
| --- | --- | --- | --- | --- | --- | --- | --- |
| XGboost | 1 | 0.28 | 0.38 | <0.01 | <0.01 | <0.01 | <0.01 |
| Logistic Regression | 0.28 | 1 | 0.15 | <0.01 | <0.01 | <0.01 | <0.01 |
| ICH-LR2S2 | 0.38 | 0.15 | 1 | <0.01 | <0.01 | <0.01 | <0.01 |
| ICH-APS | <0.01 | <0.01 | <0.01 | 1 | 0.94 | <0.01 | <0.01 |
| PASS | <0.01 | <0.01 | <0.01 | 0.94 | 1 | <0.01 | <0.01 |
| ISAN | <0.01 | <0.01 | <0.01 | <0.01 | <0.01 | 1 | <0.01 |
| PNA | <0.01 | <0.01 | <0.01 | <0.01 | <0.01 | <0.01 | 1 |


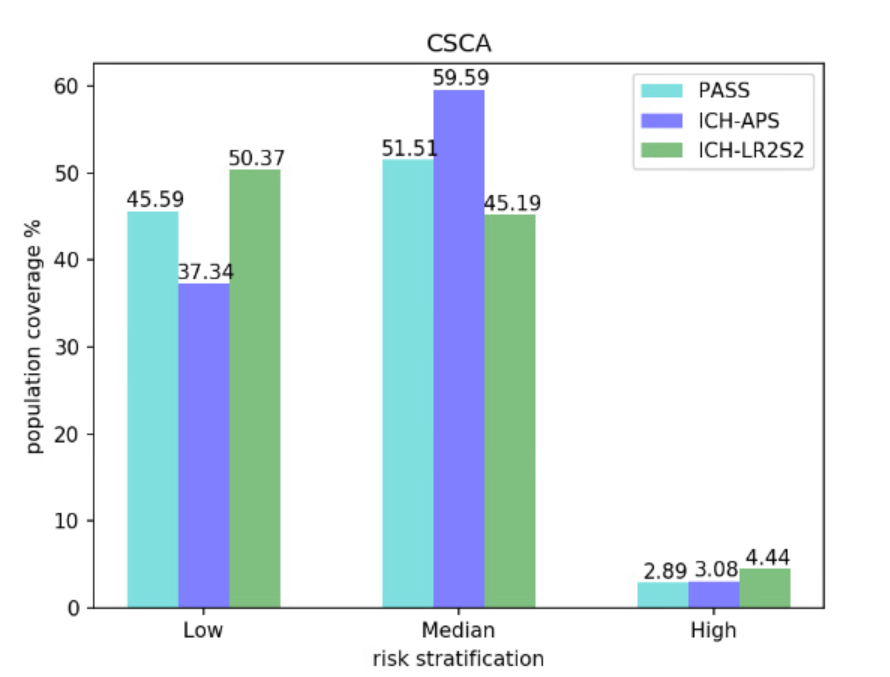


Figure S7: Pneumonia population coverage of CSCA cohort.


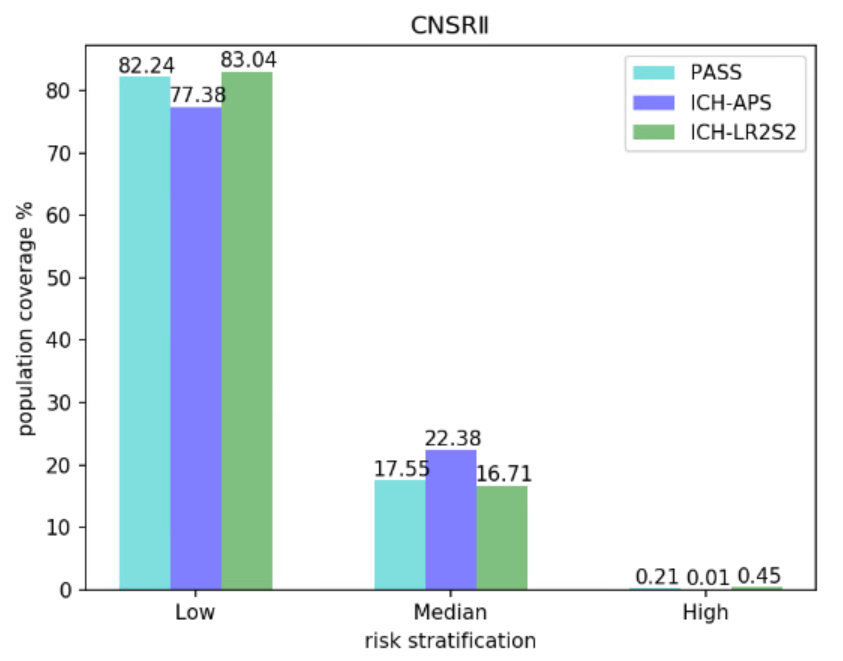


Figure S8: Pneumonia population coverage of CNSR Ⅱ cohort.


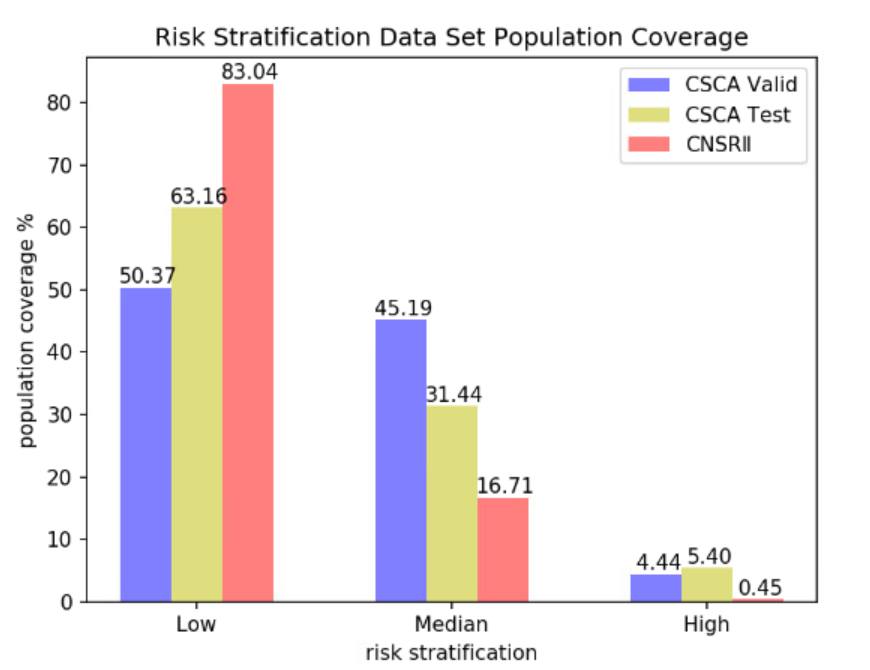


Figure S9: Comparisons of population coverage of different data sets for risk stratification.


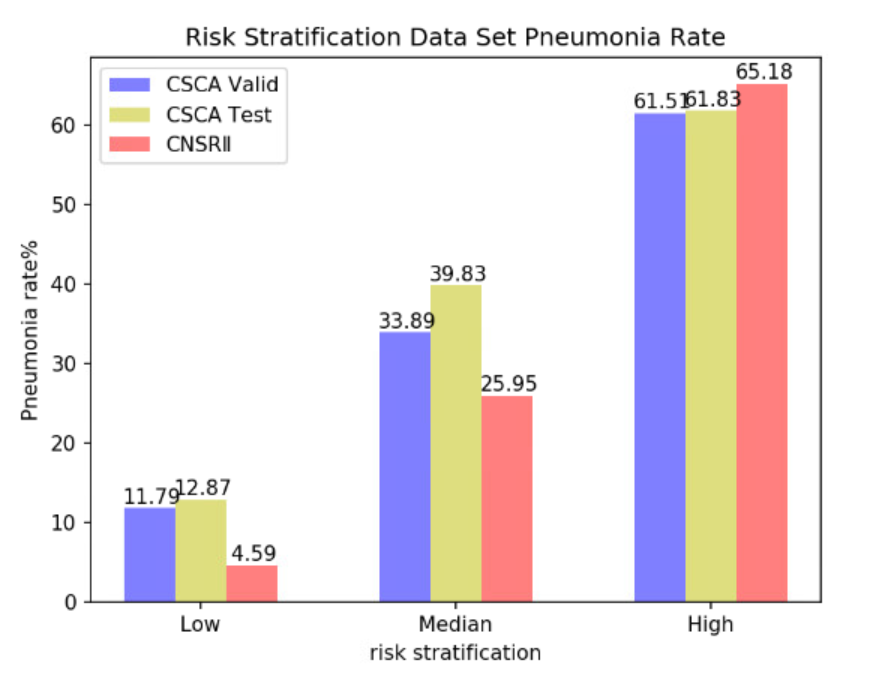


Figure S10: Comparisons of pneumonia rates in different data sets of risk stratification.
